# Nanoplasmonic immunosensor for the detection of SCG2, a candidate serum biomarker for the early diagnosis of neurodevelopmental disorder

**DOI:** 10.1038/s41598-021-02262-7

**Published:** 2021-11-23

**Authors:** So-Hee Lim, Yun-Ju Sung, Narae Jo, Na-Yoon Lee, Kyoung-Shim Kim, Da Yong Lee, Nam-Soon Kim, Jeehun Lee, Ju-Young Byun, Yong-Beom Shin, Jae-Ran Lee

**Affiliations:** 1 Rare Disease Research Center, Korea Research Institute of Bioscience and Biotechnology, 125 Gwahak-ro, Yuseong-gu, Daejeon, 34141 Korea; 2 BioNanotechnology Research Center, Korea Research Institute of Bioscience and Biotechnology, 125 Gwahak-ro, Yuseong-gu, Daejeon, 34141 Korea; 3 BioNano Health Guard Research Center (H-GUARD), 125 Gwahak-ro, Yuseong-gu, Daejeon, 34141 Korea; 4 Department of Bio-Molecular Science, KRIBB School of Bioscience, Korea University of Science and Technology (UST), 217 Gajeong-ro, Yuseong-gu, Daejeon, 34113 Korea; 5 Laboratory Animal Resource Center, Korea Research Institute of Bioscience and Biotechnology, 125 Gwahak-ro, Yuseong-gu, Daejeon, 34141 Korea; 6 Department of Pediatrics, Samsung Medical Center, Sungkyunkwan University School of Medicine, 81 Irwon-ro, Gangnam-gu, Seoul, 06351 Korea

**Keywords:** Biotechnology, Neuroscience, Biomarkers, Diseases, Medical research

## Abstract

The neural circuits of the infant brain are rapidly established near 6 months of age, but neurodevelopmental disorders can be diagnosed only at the age of 2–3 years using existing diagnostic methods. Early diagnosis is very important to alleviate life-long disability in patients through appropriate early intervention, and it is imperative to develop new diagnostic methods for early detection of neurodevelopmental disorders. We examined the serum level of secretogranin II (SCG2) in pediatric patients to evaluate its potential role as a biomarker for neurodevelopmental disorders. A plasmonic immunosensor performing an enzyme-linked immunosorbent assay (ELISA) on a gold nanodot array was developed to detect SCG2 in small volumes of serum. This nanoplasmonic immunosensor combined with tyramide signal amplification was highly sensitive to detect SCG2 in only 5 μL serum samples. The analysis using the nanoplasmonic immunosensor revealed higher serum SCG2 levels in pediatric patients with developmental delay than in the control group. Overexpression or knockdown of SCG2 in hippocampal neurons significantly attenuated dendritic arborization and synaptic formation. These results suggest that dysregulated SCG2 expression impairs neural development. In conclusion, we developed a highly sensitive nanoplasmonic immunosensor to detect serum SCG2, a candidate biomarker for the early diagnosis of neurodevelopmental disorders.

## Introduction

Neurodevelopmental disorders occur at an early stage from the neonatal period (before 1 year of age) to infancy, and adversely affect the patient’s quality of life. According to the Diagnostic and Statistical Manual of Mental Disorders, 5th Edition (DSM-5), neurodevelopmental disorders include intellectual disability, global developmental delay, communication disorders, autism spectrum disorder, attention deficit/hyperactivity disorder, specific learning disorder, and motor disorders^1^. These disorders are characterized with developmental deficits in cognition, language, behavior, and motor skills that impair personal, social, academic, and occupational functioning^2^. Early diagnosis of neurodevelopmental disorders enables early intervention, such as appropriate education or rehabilitation, to alleviate lifelong disability in patients. While neural circuits in infant brains are rapidly established around 6 months of age, the existing diagnostic methods can diagnose neurodevelopmental disorders only by the age of 2 to 3 years. Delayed diagnosis reduces therapeutic effectiveness and can have a life-time impact on the patient’s quality of life. Therefore, there is an urgent need to develop new diagnostic methods for the early detection of neurodevelopmental disorders. Disease-specific biomarkers are useful for development of scientific and systematic diagnostic tools. Next-generation sequencing (NGS) technology has allowed production of massive genomic data and identification of many genetic biomarkers^2,3^. The expression patterns of proteins reflect the biological functions of cells better than those of mRNA or DNA; thus, it is imperative to identify protein biomarkers^4,5^. A large-scale study of total protein was recently conducted to identify protein biomarkers for neurodevelopment disorders^6^. The detection of protein biomarkers can be performed using brain tissues and body fluids such as cerebral spinal fluid (CSF) or serum, but diagnosis using brain tissues is not common, except in special cases. Given the inherent risk related to the process of extracting CSF, diagnostic tools using serum are preferred for pediatric patients. However, little is known about serum biomarkers for neurodevelopmental disorders, and diagnostic methods using serum biomarkers have not been sufficiently developed.

Secretogranin II (SCG2) is a member of the chromogranin/secretogranin family of neuroendocrine secretory proteins that is largely expressed in the central nervous system and is also secreted by endocrine organs. SCG2 has been known to be involved in the packing/sorting of peptide hormones/neuropeptides into secretory vesicles. Full-length SCG2 is degraded by proteases to form seceretoneurin and chemotaxic active peptides called EM66. Although the role of SCG2 in the central nervous system is incompletely understood, SCG2 expression is known to be regulated by neurotransmitter inputs and inhibited by RE-1 silencing transcription factor (REST) binding to the promoter region of the *SCG2* gene^7,8^. Previously, SCG2 has been suggested as a CSF biomarker for multiple sclerosis, and mild cognitive impairment, pre-stage Alzheimer’s disease^9,10^. In the present study, we examined the serum level of SCG2 in pediatric patients using a nanoplasmonic immunosensor to predict its potential role as a biomarker for neurodevelopmental disorders.

Localized surface plasmon resonance (LSPR)-based sensors have been widely used in various research fields such as biology, environment, and clinical research^11–13^. LSPR occurs at the nano-sized metal structures owing to the isolated electronic oscillation^14^. Biomolecular interactions at the surface of nanostructures directly lead to local refractive index changes, eventually resulting in a red-shift of the LSPR band^15–20^. In a previous study, we detected alpha fetoprotein (AFP) in serum samples using a plasmonic immunosensor that performed an enzyme-linked immunosorbent immunoassay (ELISA) process on a gold nanodot array (GNA)^21–23^. The GNA can be easily and reproducibly fabricated by nanoimprint lithography (NIL) method that is a stamp-based process with low-cost, high-throughput, and high resolution. After the conformation of immunocomplex between antibody and antigen, the enzyme-driven catalytic reaction produces an insoluble precipitate with a high dielectric constant that is stacked on the metal surface. This approach increases the effective change in the refractive index near the metal surface and allows for the sensitive detection of molecular events occurring at the surface of metal nanostructures.

Herein, we demonstrated that a nanoplasmonic immunosensor could be employed as a biomedical diagnostic tool to quantitatively analyze serum SCG2 levels. Diagnostic methods require high sensitivity to detect biomarkers in small amounts of samples because of difficulties involved in collecting blood from pediatric patients. A strategy was developed using an enzyme precipitation reaction combined with tyramide signal amplification (TSA) to amplify the wavelength movement observed in the LSPR-based immunosensor. Tyramide is a phenolic derivative reagent that can serve as a substrate for horseradish peroxidase (HRP) in enzyme-mediated signal amplification^24,25^. HRP catalyzes the reporter-conjugated tyramine, and the deposition of active tyramine at the site of the enzyme reaction induces the accumulation of large numbers of reporter molecules that enhance the signal^26–28^. In this assay, biotin-conjugated tyramide deposited by HRP binds with streptavidin-conjugated alkaline phosphatase (AP), resulting in localized signal enhancement. The enhanced nanoplasmonic immunosensor (E-NPIS) enabled the detection of serum SCG2 in small volumes of samples to evaluate its potential as a biomarker for neurodevelopmental disorders.

## Results

### Conventional ELISA for SCG2 detection

To quantitatively analyze SCG2, a sandwich ELISA method was developed. Anti-SCG2 antibody was raised against human SCG2 peptide in rabbit and purified from the rabbit serum using an antigen peptide. Immunoblotting was performed with membrane proteins from rat brain tissues; the homemade SCG2 antibody detected SCG2 protein to a level comparable to that obtained with a commercial SCG2 antibody (Fig. S1A). In addition, the homemade SCG2 antibody worked well for immunoblotting and immunoprecipitation of recombinant human SCG2 protein expressed in heterologous cells (Fig. S1B). The homemade SCG2 antibody was employed as a capture antibody for the detection of SCG2 in the human serum using sandwich ELISA. Before constructing a standard curve for SCG2, the appropriate concentration of biotinylated SCG2 detection antibody was determined by measuring the absorbance signal at different concentrations of the detection antibody in the presence and absence of recombinant SCG2. Finally, 2.5 μg/mL was chosen as the optimal concentration of the detection antibody (Fig. S2A). The sandwich ELISA was performed under optimized conditions by varying the concentration of SCG2 diluted in 10% human serum to generate a standard curve; the absorbance exhibited linearity (R^2^ = 0.9911) corresponding to SCG2 concentration. With this ELISA method, the limit of detection (LOD), defined by the signal at zero analyte concentration plus three standard deviations, was 5.2 ng/mL SCG2 (Fig. S2B).

### Nanoplasmonic immunosensor for SCG2 detection

**Figure 1:**
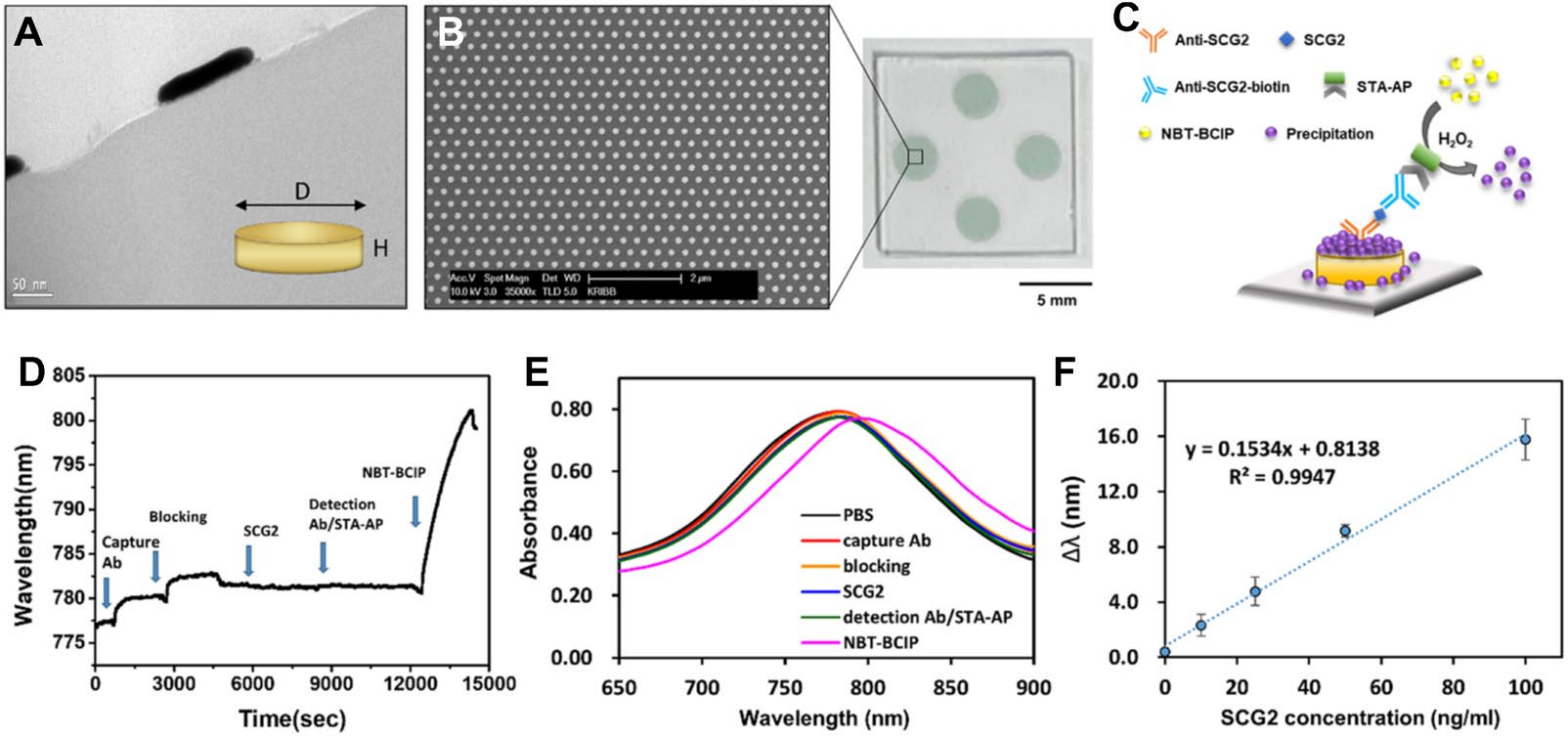
Nanoplasmonic immunosensor for detection of serum SCG2. (**A**) Transmission electron microscope (TEM) image of a gold nanodot. D (diameter): 150 nm, H (height) 20 nm. (**B**) Scanning electron microscope (SEM) image of gold nanodot array (GNA) uniformly fabricated on a glass substrate. The GNA chip has a size of 13 mm × 13 mm. The size of the circle sensing region inside the chip is 3 mm in diameter. (**C**) A schematic representation of the nanoplasmonic immunosensor using enzyme catalytic precipitation reaction. STA-AP, streptavidin–alkaline phosphatase-conjugate. The real-time sensorgram (**D**) and LSPR spectral changes (**E**) of each step in the immunoassay for SCG2 (100 ng/mL). (**F**) Standard curve was prepared by plotting the ∆λ measured with varying concentration of SCG2 (from 0 to 100 ng/mL). The regression equation was y = 0.1534x + 0.8138 (R^2^ = 0.9947) in the linear range. Each data point is the average of N = 3 individual measurements, and the error bars indicate standard deviation.

Then, we developed a plasmonic immunosensor combined with enzyme-based precipitation method to quantitatively analyze SCG2 with high sensitivity in small volumes of infant serum samples. Gold nanodot array (GNA) was used as the plasmonic material; the GNA chips were easily fabricated using UV nanoimprint lithography. As shown in Fig. 1A, B cross-sectional transmission electron microscopy (TEM) image of the GNA chip revealed a gold nanodot on a glass wafer with a diameter and height of 150 and 20 nm, respectively. Figure 1B shows that the hexagonal array of GNA was uniformly fabricated over a large area, and four nanodot-patterned areas were prepared on a single chip for multiple detection. Following the fabrication process, the GNA surface was functionalized with thiol groups by a self-assembly monolayer (SAM) reaction for antibody immobilization (Fig. S3). Immobilization of SCG2 capture antibody onto the GNA chip resulted in a red shift of the LSPR band from 777.1 to 779.6 nm, indicating that the antibody was immobilized onto the surface of GNA. Following a blocking step, SCG2-spiked human serum samples were incubated above the sensor chip arrayed with the SCG2 capture antibody. After detection with a biotinylated antibody, the streptavidin–alkaline phosphatase (STA–AP) conjugate was attached to the immunocomplex on GNA surface. The enzyme-mediated reaction catalyzed the conversion of multiple substrate molecules (NBT-BCIP, nitro blue tetrazolium & 5-bromo-4-chloro-3-indolyl phosphate p-toluidine) into precipitates (Fig. 1C). Figure 1D displays a sensorgram that demonstrates the changes in the LSPR wavelength monitored in real time corresponding to each binding process. Figure 1E shows the LSPR spectra of the GNA after each stage in the stepwise procedure for Fig. 1D. The LSPR peak shift (∆λ was defined as the shift in LSPR wavelength between before and after NBT-BCIP reaction) increased from 780.9 to 799.2 nm after the enzyme-mediated precipitation reaction on the GNA chip. The generated insoluble precipitate eventually induced a large shift in the LSPR peak to a longer wavelength region. The titration experiments to detect SCG2 in 10% human serum revealed that the LOD was 0.290 ng/mL and the slope of the LSPR shift was linear (R^2^ = 0.9947) as the SCG2 concentration increased in the range of 0–100 ng/mL (Fig. 1F). In comparison with the conventional ELISA, the nanoplasmonic immunosensor showed approximately 18-fold higher sensitivity.

### SCG2 detection with nanoplasmonic immunosensor combined with TSA

**Figure 2:**
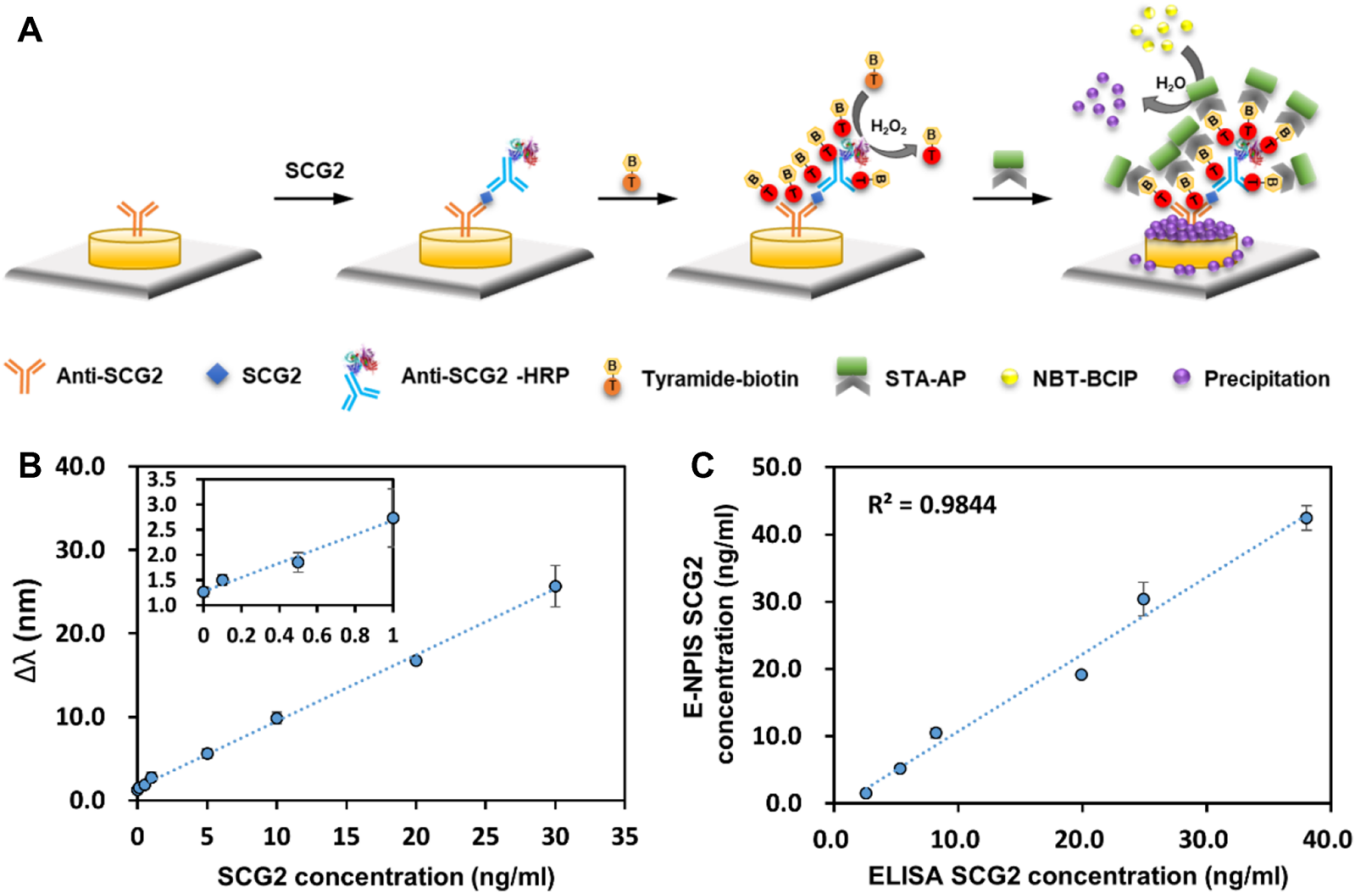
Enhanced nanoplasmonic immunosensor based on tyramide amplification strategy. (**A**) Schematic illustration of the enhanced nanoplasmonic immunosensor using enzyme precipitation reaction combined with tyramide signal amplification for signal enhancement. After immunoreaction is completed, the tyramide-biotin conjugates were deposited by HRP (horseradish peroxidase) catalyzed reaction. A subsequent reaction with STA-AP results in the localization of the enhancement of the AP signal at the site of tyramide deposition. (**B**) Standard curve was prepared by plotting the ∆λ measured with varying concentration of SCG2 (from 0 to 30 ng/mL). The inset plot shows the assay response in the low concentration area at below 1.0 ng/mL. The regression equation was y = 0.7947x + 1.5427 (R^2^ = 0.9983) in the linear range. (**C**) The correlation between enhanced nanoplasmonic immunosensor (E-NPIS) and conventional ELISA for the detection of SCG2 (^2^ R^2^ = 0.9844). Each data point is the average of N = 3 individual measurements, and the error bars indicate standard deviation.

To enhance the signal generated from the AP-mediated reaction, the nanoplasmonic immunosensor was optimized using the tyramide signal amplification (TSA) strategy. The schematic representation explains the signal enhancement mechanism of the nanoplasmonic immunosensor for SCG2 detection using the TSA system (Fig. 2A). After the binding of the biotinylated detection antibody to the captured SCG2, streptavidin–horseradish peroxidase (STA-HRP) gets attached to the immunocomplex. HRP then catalyzes the deposition of biotin-tyramide in the presence of hydrogen peroxide. This reaction covers the immunocomplex surface with biotin in large amounts for detection by STA-AP. The NBT-BCIP precipitate formed by AP is stacked on the GNA surface, consequently resulting in a large increase in the local dielectric constant.

Before constructing the standard curve for SCG2, we optimized the blocking solution condition to minimize any non-specific binding between biotinylated detection antibodies and STA-HRP to achieve high signal-to-background ratios. The lowest LSPR peak shift in the absence of target molecules illustrated the successful inhibition of nonspecific binding by 5% skim milk to obtain the best sensitivity and LOD (Fig. S4A). Next, the appropriate concentration of biotinylated SCG2 detection antibody was determined by measuring the LSPR peak shift in the presence and absence of SCG2. As TSA is based on the enzyme-driven substrate deposition, the amount of HRP attached to the biotinylated detection antibody is critical for the analytical performance of the assay. High concentration of antibody can lead to relatively high steric hindrance, thereby preventing the biotin-tyramide and STA-AP reaction. On the other hand, low HRP concentration may be insufficient to catalyze the deposition of biotin-tyramide molecules and reduce the reaction sensitivity. As the LSPR peak shift increased until 5 μg/mL concentration of biotinylated detection antibody and reached a saturation point thereafter, we chose this value as the optimal concentration of detection antibody for further assays (Fig. S4B).

We evaluated the sensitivity and quantitative performance of the proposed method for SCG2 analysis under optimal conditions. The standard curve for SCG2 was established in the concentration range of 0–30 ng/mL (Fig. 2B). Recombinant SCG2 protein was spiked into 10% human serum at final concentrations representative of clinical test samples. The LOD of the enhanced nanoplasmonic immunosensor (E-NPIS) for SCG2 was 0.016 ng/mL, which was 325-fold lower than that of the conventional ELISA. To confirm the specificity of the proposed assay for the detection of SCG2 in human serum, we analyzed specificity using several serum proteins including IL-6 (interluekin-6), PSA (prostate specific antigen), and AFP (alpha fetoprotein). As shown in Fig. S5, a large peak shift was observed only in the presence of SCG2 in 10% human serum. The LSPR peak shifts were comparable to the background signal in the presence of other interfering substances. These results indicated that the proposed assay exhibited good performance for discriminating SCG2 from possible interfering substances. We also confirmed that the results of the enhanced nanoplasmonic immunosensor matched those of the conventional ELISA (Fig. 2C). A good correlation between ELISA (x-axis) and enhanced nanoplasmonic immunosensor (y-axis) was obtained with the linear regression equation y = 1.1471x − 0.7453 (R^2^ = 0.9844). These results demonstrate that the proposed method could accurately detect SCG2 in serum samples.

### Detection of SCG2 in clinical samples using enhanced nanoplasmonic immunosensor

**Figure 3:**
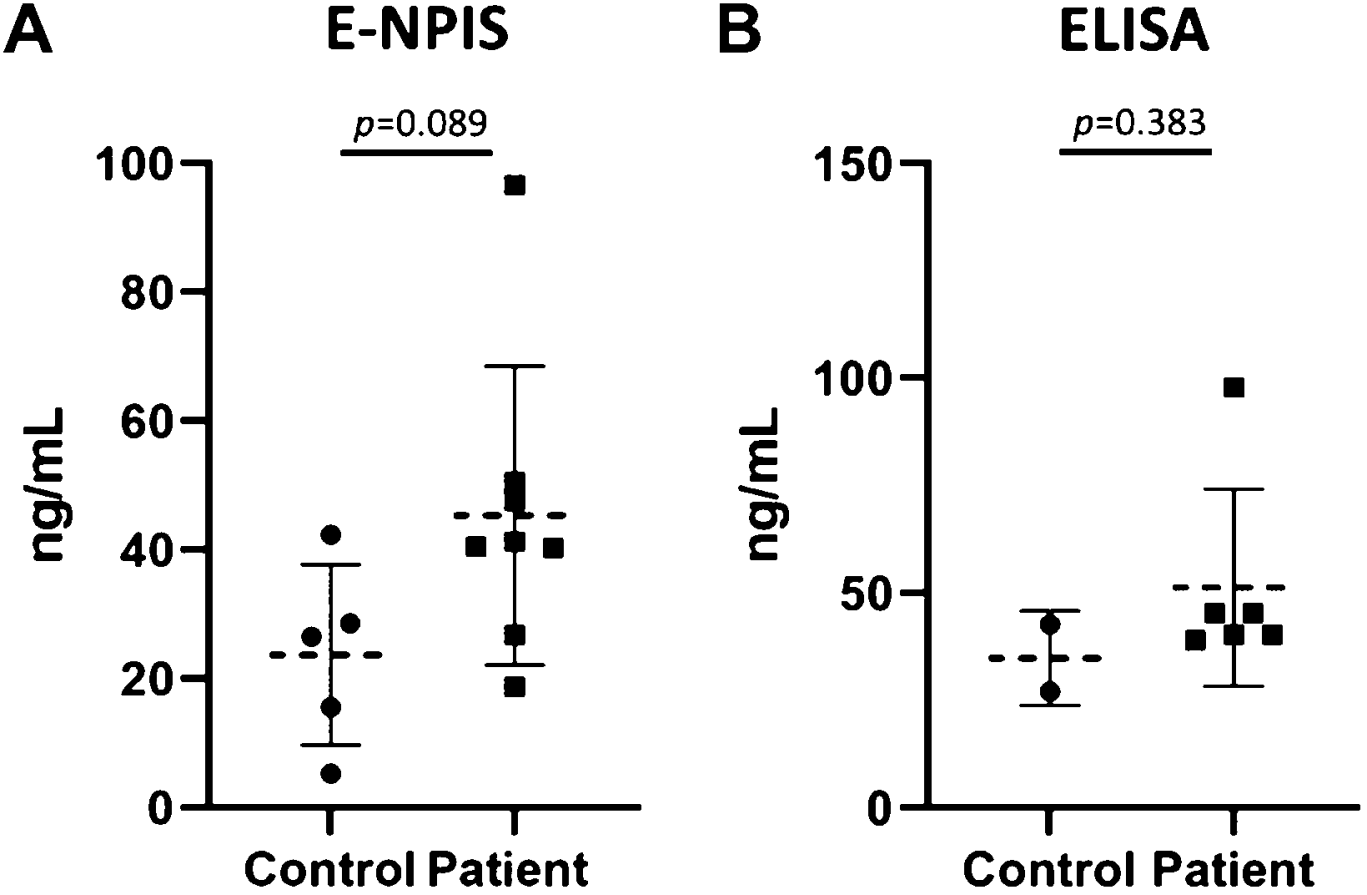
Analysis of SCG2 concentration in clinical serum samples. SCG2 concentration in serum samples of patients and control groups was analyzed with enhanced nanoplasmonic immunosensor (E-NPIS) (**A**) and ELISA (**B**). The serum samples were 1:10 diluted (E-NPIS) or 1:5 diluted (ELISA) with PBS buffer. Data represent the means ± s.d. of three replicates from 5 controls and 8 patients for E-NPIS analysis, 2 controls and 6 patients for ELISA analysis. SCG2 concentration was below the LOD of ELISA for some clinical samples. *P* = 0.089 for E-NPIS and *p* = 0.383 for ELISA (Student’s t-test). Basic information of clinical serum samples was described in Table S1 (Supplementary information).

Finally, we validated the feasibility of the enhanced nanoplasmonic immunosensor (E-NPIS) for the detection of SCG2 in clinical samples from pediatric patients. Whole serum samples (5 μL) of eight patients with developmental delay and five controls were 1:10 diluted in a buffer for measurement of SCG2 using the E-NPIS (Table S1 and Fig. 3A). SCG2 was successfully detected in the tenfold-diluted serum samples from pediatric patients using E-NPIS in the concentration range of 1.88 to 9.66 ng/mL (average of 4.54 ng/mL, corresponding to 45.4 ng/mL in whole serum). The concentrations of SCG2 in the tenfold-diluted serum samples from the control group were between 0.53 and 4.24 ng/mL (average of 2.37 ng/mL, corresponding to 23.7 ng/mL in whole serum). The E-NPIS effectively detected SCG2 at low concentrations, which were undetectable using ELISA (LOD, 5.2 ng/mL). Thus, the sensitivity of the proposed method was sufficient to quantify SCG2 in small volumes of serum samples from pediatric patients. As shown in Fig. 3A, the levels of SCG2 were higher in the developmental delay group than in the control group, although the difference was not statistically significant (*p* = 0.089). When the serum samples were 1:5 diluted and analyzed using the conventional ELISA method, the patient group displayed higher SCG2 concentrations than the control group; this result was similar to that observed with the E-NPIS (Fig. 3B). The conventional ELISA method allowed SCG2 detection in the concentration range of 7.79 to 19.55 ng/mL (average of 10.26 ng/mL, corresponding to 51.3 ng/mL in whole serum) in fivefold-diluted serum samples from the patient group and 5.41 to 8.53 ng/mL (average of 6.97 ng/mL, corresponding to 34.9 ng/mL in whole serum) in fivefold-diluted serum samples from the control group. In conclusion, this highly sensitive nanoplasmonic immunosensor enabled the detection of SCG2 in small volumes of serum, and revealed the higher serum SCG2 levels in patients with developmental delay.

### Regulation of neuronal development by SCG2

**Figure 4:**
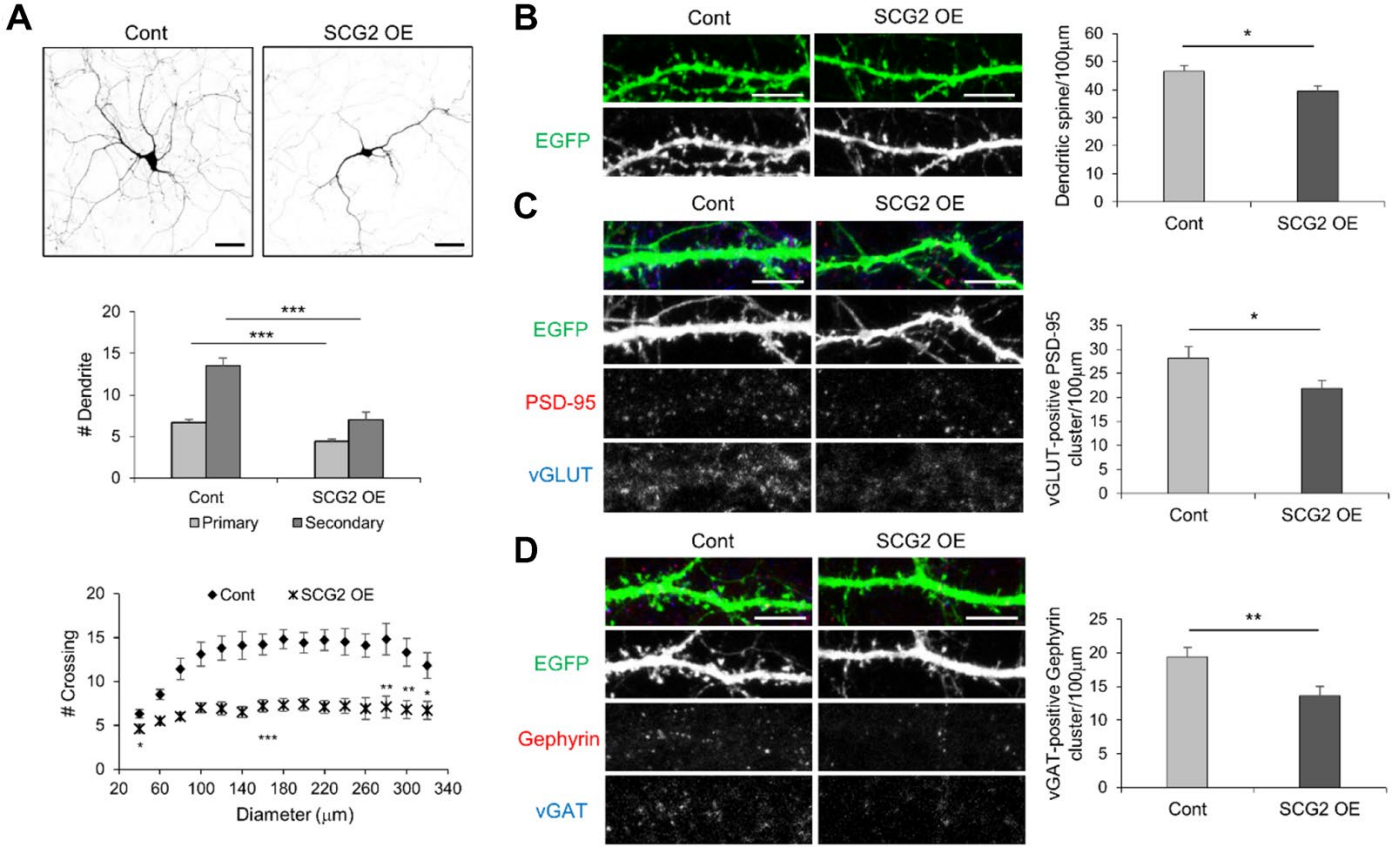
Dendritic arborization and synaptic formation attenuated by SCG2 overexpression. (**A**) Attenuated dendritic arborization by SCG2 overexpression. Rat hippocampal neurons were transfected with human SCG2 clone (hSCG2) (Fig. S1B). The numbers of dendrites and arborization significantly decreased after SCG2 overexpression (SCG2 OE) as compared with that in the control group that expressed EGFP only (Cont). Means $\pm$ s.e.m. of data from 10 control neurons (Cont) and 10 hSCG2 neurons (SCG2 OE) are shown. For primary dendrites: ***p < 0.001; for secondary dendrites: ***p < 0.001; for Sholl analysis of arborization: *p < 0.05, **p < 0.01, and ***p < 0.001 (Student’s t-test). Scale bar: 50 μm. (**B–D**) Decreased synaptic formation by overexpression of SCG2. The density of dendritic spines and the numbers of excitatory and inhibitory synapses significantly decreased after SCG2 overexpression (SCG2 OE) as compared with that in the control group (Cont). Means $\pm$ s.e.m. of data from 28 dendrites of 7 control neurons (Cont) and 28 dendrites of 7 hSCG2 neurons (SCG2 OE). (**B**) The density of dendritic spines: *p < 0.05. (**C**) The numbers of excitatory synapses: *p < 0.05. (**D**) The numbers of inhibitory synapses: **p < 0.01 (Student’s t-test). Scale bar: 10 μm.

Whether SCG2 expression is closely associated with neuronal development or neurological function is questionable; thus, we examined the relationship between SCG2 expression and neuronal development (Fig. 4). We transfected hippocampal neurons with a human SCG2 expression construct. Primary/secondary dendritic analysis and Sholl profile revealed the attenuation of dendritic arborization in SCG2 overexpression (SCG2 OE) group as compared with that in the EGFP expression group (Cont) (Fig. 4A). In addition, the density of dendritic spines and the number of excitatory (puncta of vGLUT-positive PSD-95) and inhibitory (puncta of vGAT-positive gephyrin) synapses decreased after SCG2 overexpression as compared with that in the control group (Fig. 4B–D). These results show that neuronal development is attenuated by SCG2 overexpression, and that the dysregulation of SCG2 expression could be a cause of neurodevelopmental disorders.

**Figure 5:**
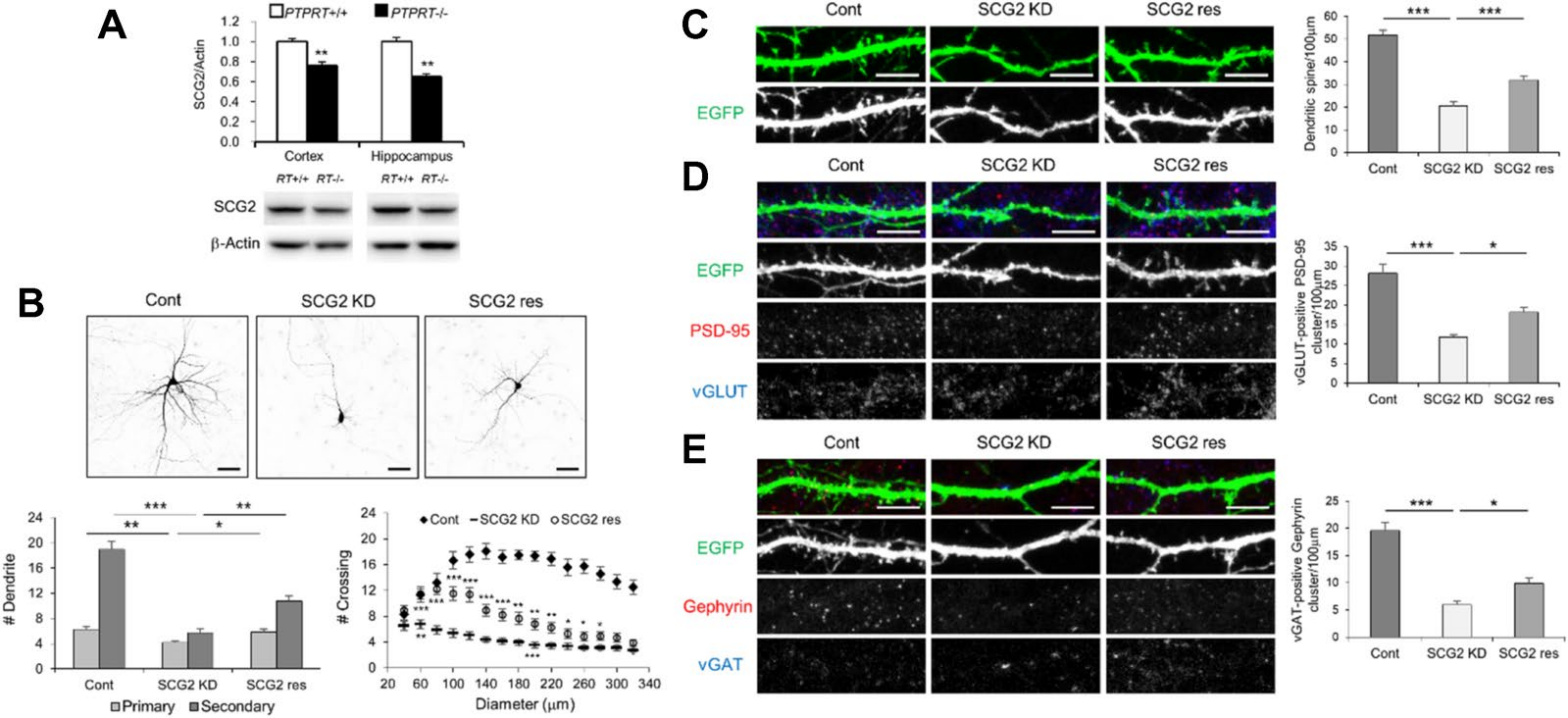
Dendritic arborization and synaptic formation attenuated by SCG2 knockdown. (**A**) Decreased SCG2 in the brain tissues of *PTPRT*^−/−^ null mice. SCG2 expression was examined in the cortical and hippocampal tissues of *PTPRT*^−/−^ null mice. Western blotting displayed that SCG2 levels relative to β-actin decreased in *PTPRT-/-* null mice as compared with wild-type mice (*n* = 7, 6). **p < 0.01 (Student’s t-test). The original images are presented in Supplementary Fig. S6. (**B**) Attenuated dendritic arborization by SCG2 knockdown. Rat hippocampal neurons were transfected with SCG2-shRNA to knock down SCG2 expression (Fig. S7A). The numbers of dendrites and arborization significantly decreased by SCG2 knockdown (SCG2 KD) as compared with the control group (Cont), and were rescued by the addition of Res SCG2 resistant to SCG2-shRNA to this context (SCG2 res) (Fig. S7B). Means $\pm$ s.e.m. of data from 12 control neurons (Cont), 12 SCG2-shRNA neurons (SCG2 KD), and 14 SCG2-shRNA + Res SCG2 neurons (SCG2 res) are shown. By the Turkey post hoc test after application of one-way ANOVA, primary dendrites: *p < 0.05 and **p < 0.01, *F* = 6.887, *p* = 0.003; secondary dendrites: **p < 0.01 and ***p < 0.001, *F* = 45.80, *p* < 0.0001. Sholl analysis of arborization: *p < 0.05, **p < 0.01, and ***p < 0.001 (Student’s t-test). Scale bar: 50 μm. (**C–E**) Decreased synaptic formation by SCG2 knockdown. The density of dendritic spines and the numbers of excitatory and inhibitory synapses significantly decreased by SCG2 knockdown (SCG2 KD) as compared with the control group (Cont), and were rescued by the addition of Res SCG2 to this context (SCG2 res). Means $\pm$ s.e.m. of data from 28 dendrites of 7 control neurons (Cont), 26 dendrites of 7 SCG2-shRNA neurons (SCG2 KD), and 26 dendrites of 7 SCG2-shRNA + Res SCG2 neurons (SCG2 res) are shown. By the Turkey post hoc test after application of one-way ANOVA, the density of dendritic spines: ***p < 0.001, *F* = 66.67, *p* < 0.0001; the numbers of excitatory synapses: *p < 0.05 and ***p < 0.001, *F* = 24.67, *p* < 0.0001; the numbers of inhibitory synapses: *p < 0.05 and ***p < 0.001; *F* = 37.65, *p* < 0.0001. Scale bar: 10 μm.

We then examined SCG2 expression in a *PTPRT*^−/−^ null mouse model of developmental delay. Protein tyrosine phosphatase receptor T (PTPRT) is principally expressed in the central nervous system and regulates neuronal development^29,30^. *PTPRT* gene abnormalities induce neurological diseases such as intellectual disability, autism, and depression^31–34^. *PTPRT*^−/−^ null mice were shown to exhibit attenuated synaptic formation and synaptic plasticity as well as reduced pluripotency of neural stem cells in the hippocampus^30^. In the present study, we found that SCG2 expression decreased in the cortical and hippocampal tissues of *PTPRT*^−/−^ null mice as compared with that in the wild-type mice (Fig. 5A). Therefore, we examined whether neuronal development of hippocampal neurons was attenuated by reduced SCG2 expression. When we knocked down SCG2 expression using SCG2-shRNA (SCG2 KD), dendritic arborization of hippocampal neurons was attenuated as compared with the control (Cont) group, and was rescued by SCG2 construct resistant to SCG2-shRNA (SCG2 res) (Fig. 5B). The density of dendritic spines, and the numbers of excitatory and inhibitory synapses decreased after SCG2 knockdown as compared with control, and were rescued by SCG2 construct resistant to SCG2-shRNA (Fig. 5C–E). Thus, the appropriate expression level of SCG2 contributes to normal neurodevelopment, and that any dysregulation in SCG2 expression may induce neurodevelopmental disorders. In this respect, the altered SCG2 concentration could serve as a diagnostic indicator for impaired neurodevelopment; thus, we recommend SCG2 as a candidate serum biomarker of neurodevelopmental disorders.

## Discussion

The prevalence of neurodevelopmental disorders exceeds ~ 17% in children between 3 and 17 years of age. These disorders are the most prevalent chronic medical conditions demanding pediatric primary care^1,35^. The diagnosis of neurodevelopmental disorders is mainly established based on methods dependent on language, communication, social interaction, facial expression, and intelligence quotient test. These methods pose difficulties to diagnose neurodevelopmental disorders at an early stage, except for some severe developmental disabilities. A child with global developmental delay typically presents with a delay in multiple developmental areas such as speech, language, social, cognition, play, and motor skills. The diagnosis of global developmental delay is limited to children younger than 3–5 years of age, but these children often evolve to meet the diagnostic criteria for intellectual disability. Here, we developed a new diagnostic method using serum biomarkers for infant patients to overcome the limitations of current diagnostic methods. For the detection of SCG2, a candidate serum biomarker of neurodevelopmental disorder, the nanoplasmonic immunosensor employs an enzyme precipitation reaction combined with a tyramide amplification strategy. This enhanced nanoplasmonic immunosensor (E-NPIS) achieved a very low LOD and was approximately 325 times more sensitive than the conventional ELISA method. The performance of the enhanced nanoplasmonic immunosensor is more than comparable to other LSPR based immunosensors (Table S2). With only 5 μL clinical serum samples, the E-NPIS revealed the increase in SCG2 concentration in patients with developmental delay. On the other hand, 5 μL of clinical serum sample was not sufficient for conventional ELISA because SCG2 concentration was below its LOD. As the amount of serum sample was doubled to 10 μL in conventional ELISA, SCG2 concentration was still below the LOD for some clinical samples. Although the number of samples was not sufficient to draw conclusions, the analysis with conventional ELISA showed that serum SCG2 level increased in the patients as compared with that in the control group. These results suggest that SCG2 could be used as a serum biomarker for the early diagnosis of neurodevelopmental disorders. The reason the difference in SCG2 levels between patients and controls was not statistically significant could be that the sample number was too small or that serum SCG2 concentration inherently did not change much. In previous study, there has been not much difference in the serum SCG2 concentration between patients with Parkinson’s disease and controls compared to other biomarkers^36^.

Various metabolites, amino acids, peptides, and proteins have been recently identified as potential biomarkers for neurodevelopmental disorders^37–39^. The blood–brain barrier (BBB) defends the central nervous system by limiting the entry of harmful molecules from the bloodstream into the brain. Therefore, BBB dysfunction is a cause of many neurological diseases, and neuronal proteins secreted into the blood through the damaged BBB can be used as useful biomarkers^4^. SCG2 is expressed in large quantities in the central nervous system and is located mainly in the basal ganglia, hypothalamus, and pituitary gland. Seceretoneurin, a degraded peptide of SCG2, has been found to promote neurite outgrowth in immature cerebellar granule cells^40^. A previous study using mass spectrometry showed that the concentration of SCG2 decreased in the CSF of patients with multiple sclerosis, and on the contrary, increased in those with mild cognitive impairment, a pre-stage Alzheimer’s disease^9,10^. In our study, the patients with developmental delay showed an increase in the serum level of SCG2 as compared with the control group. Interestingly, SCG2 expression reduced in the brain tissue of an animal model of developmental delay that showed attenuated synaptic plasticity and aberrant neurogenesis^30^. In hippocampal neurons, dendritic arborization and synaptic formation were attenuated by overexpression or knockdown of SCG2. SCG2 is involved in the packing/sorting of peptide hormones/neuropeptides, and its expression is regulated by neurotransmitter inputs; however, the exact role of SCG2 is not well understood. Learning and memory require the formation of new neural networks in the brain, and the key mechanism underlying this process is synaptic plasticity^41^. Synaptic plasticity is a process by which the strength of synaptic transmission is altered in response to the pattern of synaptic activity. Neurotransmitter inputs regulate gene expression, which underlies long-term forms of learning and memory^7^. SCG2 expression is activated by in-phase inputs of neurotransmitters and, thus, SCG2 has been suggested as a signal integrator of synaptic transmission. Therefore, SCG2 appears to play a crucial role in learning and memory, and dysregulated SCG2 expression could induce neurodevelopmental deficits; further studies with *SCG2* gene-modified animals are warranted to confirm these hypotheses. In conclusion, we developed a highly sensitive nanoplasmonic immunosensor for the detection of serum SCG2, which could be used for the early diagnosis of neurodevelopmental disorders such as global developmental delay.

## Methods

### Materials

11-Mercaptoundecanoic acid (MUA), 1-ethyl-3-(3-dimethylaminopropyl)-carbodiimide (EDC), N-hydroxysuccinimide (NHS), bovine serum albumin (BSA), human serum, streptavidin–alkaline phosphatase (STA-AP), streptavidin–horseradish peroxidase (STA-HRP), nitro blue tetrazolium (NBT), and 5-bromo-4-chloro-3-indolyl phosphate p-toluidine (BCIP) were purchased from Sigma-Aldrich (St. Louis, MO, USA). Skim milk was purchased from BD Difco (Franklin Lakes, NJ, USA). UV-curable perfluoropolyether (PFPE), which is used for replication of the master and imprint resin, and mr-I PMMA 35k200 were purchased from Solvay Solexis (Marshalton, DE, USA) and Micro Resist Technology (Berlin, Germany), respectively. Biotin XX Tyramide SuperBoost kit was purchased from Thermo Fisher Scientific (Waltham, MA, USA). Recombinant SCG2 (Cat# MBS2028995), biotinylated anti-SCG2 antibody (Cat# MBS7046117) were obtained from MyBioSource (San Diego, USA). Scg2 capture antibody was manufactured as below: anti-SCG2 polyclonal antibody was raised against the peptide AHKEESSPDYNPYQG (human SCG2, aa 69–83) as an antigen in rabbit and purified from rabbit serum using antigen peptide covalently linked with Sulfolink coupling gel (Thermo Fisher Scientific). Other antibodies were purchased from commercial sources: rabbit anti-SCG2 (Cat# ab126935, Abcam, Cambridge, UK), mouse anti-PSD-95, rabbit anti-vGlut1 (Cat# MA1-045, Thermo Fisher Scientific), mouse anti-gephyrin (Cat# 147 111, SYSY, Goettingen, Germany), rabbit anti-vGAT (Cat# 131 002, SYSY). The Cy3, FITC, and HRP-conjugated secondary antibodies were obtained from Jackson ImmunoResearch (West Glove, PA, USA).

### Clinical human serum samples

An information sheet describing the rationale of the study and individual rights was handed to the parents of the participants. Written informed consents were then obtained for the blood samples and all experiments were performed in accordance with the approved guidelines and regulations of Human Ethics Committee and the Institutional Review Board (IRB) of Samsung Medical Center (IRB number: 01–201,604-31–003). All patients were diagnosed with global developmental delay according the guidelines of Diagnostic and Statistical Manual of Mental Disorders: Fourth Edition-V (DSM-V)^1^. The whole blood collected from participants was allowed to clot at room temperature (RT), and then the clot was removed by centrifuging at 2,000 × g for 10 min in a refrigerated centrifuge.

### SCG2 detection using conventional ELISA

The conventional ELISA method was used as the standard in this study. Typically, 100 µL of the homemade anti SCG2 antibody (2 μg/mL) was coated in 96-well plates overnight at 4 °C, and then the wells were blocked with skim milk (w/v, 5%) for 2 h at RT. After blocking, the wells were washed with PBST (PBS at pH 7.4 containing 0.1% Tween20) to remove excess skim milk. Next, 50 μL of various concentrations of SCG2 in human serum were added to the wells. The patient serums were diluted fivefold with PBS buffer. The wells were incubated at RT for 2 h and washed thrice. Later, 100 μL of biotinylated anti-SCG2 antibody solution was added to each well and incubated at RT for 1 h. The wells were washed again, and 100 μL of STA-HRP (500 ng/mL) was added to the wells and incubated at RT for 30 min. Then, the wells were washed with PBST four times, and 100 μL of TMB substrates were introduced and incubated at RT for 30 min. Finally, 50 μL of stop solution (2 M H_2_SO_4_) was added, and the optical density was measured at 450 nm using a microplate reader.

### Sensor chip fabrication

The sensor chip fabrication procedure was reported in our previously works^21^. Briefly, in order to make a sensor chip, the silicon master with the nano-hole pattern used as the master mold had a diameter, pitch, and depth of 150 nm, 300 nm, and 150 nm, respectively. The master surface was covered with a polycarbonate (PC) film after the UV resin (PFPE) to be used for the replica mold was dropped onto the surface. The PC film of the master was pressed with a hand roller to fill the hole pattern with resin, and then the replica mold was cured on the PC film. A thermoplastic resin (PMMA) was spin-coated on a glass substrate for 2 h at 130 °C (5 bar). The replica mold was separated from the substrate after cooling to 90 °C. To protect the top region of the imprinted resin from O_2_ plasma etching, a 30 nm layer of titanium was deposited at a tilted angle of 70° during rotation in an e-beam evaporator. The residual layer was removed by O_2_ plasma etching (PINK GmbH Plasma-finish, Bestenheid, Germany) at an O_2_ flow rate of 300 mL/min and power of 300 W for 6 min. Finally, a Ti/Au layer (1/20 nm) was deposited by e-beam evaporation, and lift-off was carried out using an acetone solution for 5 min. There are four regions with a diameter of 3 mm in one chip; one of the four regions was used as the reference channel, and the remaining three regions were used as the sample channels.

### SCG2 detection by signal enhanced nanoplasmonic immunosensor

The fabricated gold nanodot array (GNA) chips were cleaned with piranha solution (H_2_SO_4_/H_2_O_2_ = 3:1) at 90 °C to eliminate the impurities on the surface of the chips. The chips were washed with deionized water (DW) and dried with nitrogen gas. The cleaned chips were immersed in a 10 mM 11-mercaptoundecanoic acid (MUA) solution for 12 h. The carboxylate groups of the MUA surfaces were activated in a solution of 0.1 M 1-ethyl-3-(3-dimethylaminopropyl)-carbodiimide and 0.025 M of N-hydroxysuccinimide in DW for 9 min. Then, 0.1 mg/mL anti-SCG2 capture antibody in PBS was added to the GNA chip and incubated at 4 °C overnight. After washing the chip 3 times with 50 μL PBST, blocking solution (5% skim milk) was applied for blocking nonspecific binding, followed by incubation at 37 °C for 30 min. 50 μL of recombinant SCG2 at various concentrations (0–40 ng/mL) in human serum was injected, followed by incubation at 37 °C for 1 h. After incubation, the chips were washed with 3 times with PBST. For nanoplasmonic immunosensor, 5 μg/mL of biotinylated anti-SCG2 antibody and 5 μg/mL STA-AP were added to the chip. After the incubation for 1 h, the excess detection antibody and STA-AP were removed by washing the chips 3 times with washing buffer. Next, the enzyme-catalyzed precipitation reaction was triggered by adding 0.1 M 5-bromo-4-chloro-3-indolyl phosphate p-toluidine (BCIP) and 1 mg/mL nitro blue tetrazolium (NBT) in an AP buffer (100 mM Tris–HCl, 100 mM NaCl, 5 mM MgCl_2_) at pH 9.5 for 30 min. For enhanced nanoplasmonic immunosensor, 5 μg/mL of biotinylated anti-SCG2 antibody and 5 μg/mL of STA-HRP instead of STA-AP were added to the GNA chip. The chip was washed, and then 1 × biotin-tyramine and 0.03% H_2_O_2_ in 0.1 M borate buffer (pH 8.5) were added to the chip followed by keeping at room temperature for 15 min. After washing, 5 μg/mL of STA-AP was added to the chip and incubated at 25 °C for 30 min. Unbound STA-AP was removed by washing the chips 3 times with PBST. The enzyme-catalyzed precipitation reaction was triggered by adding 0.1 M BCIP and 1 mg/mL NBT in an AP buffer at pH 9.5 for 30 min. LSPR wavelength change was measured by LSPR observation setup with the back-reflection mode and determined by the centroid fit using a homemade program based on LabVIEW^21^. The scan area was the 1 mm diameter part located in the center of each region, and it was passed through the light source as described previously^21^.

### DNA constructs

For knockdown of rat SCG2 (NM_022669.2), nt 530–547 (GGTTCCCTCTCATGTATG, SCG2-shRNA) was subcloned into pSuper.gfp/neo (OligoEngine, Seattle, WA, USA). Full-length human SCG2 (NM_003469, aa 1–617) was subcloned into GW1-CMV, and used as Rescue SCG2 for SCG2-shRNA.

### Immunoblot analysis

Immunoblotting was performed as previously described^42^. This study was performed in accordance with the regulations outlined by the Korean law. The animal experiment protocols were approved by the Animal Use and Care Committee of Korea Research Institute of Bioscience and Biotechnology (Permit Number: KRIBB-AEC-19115). Animals were sacrificed using CO_2_ gas, and all efforts were made to minimize suffering. Briefly, mice were sacrificed and the brain tissue was quickly removed and homogenized in a homogenization buffer (50 mM Tris–HCl, 150 mM NaCl, 1% Nonidet P-40, 0.1% SDS, and 0.1% sodium deoxycholate, pH 8.0) containing protease inhibitor cocktail (Roche, Mannheim, Germany). Protein samples were resolved with SDS-PAGE and then transferred onto a polyvinylidene fluoride membrane (BioRad Laboratories, CA, USA). Blots were incubated with primary and secondary antibodies followed by visualization using an Enhanced Chemiluminescence kit (Atto Corp., Japan). Immunoblot images were quantified using Quantity One 1-D analysis version 4.6.1 software (Bio-Rad Laboratories) or Image J software (NIH) (https://imagej.nih.gov/ij/index.html).

### Transfection of neurons and immunocytochemistry

Primary hippocampal neurons were prepared from embryonic day 18 rats as described previously^29^. Briefly, hippocampi were dissected with trypsin and plated on coverslip coated with poly-L-lysine in Neurobasal medium (Thermo Fisher Scientific) supplemented with B27 (Thermo Fisher Scientific). After 2–3 h of incubation, the plating medium was changed with a growth medium (plating medium and glutamate). Cultured hippocampal neurons were transfected by the calcium phosphate method at 5 or 7 days in vitro. For immunofluorescent staining, after 7–9 days of transfection, cultured hippocampal neurons were fixed in 4% (v/v) formaldehyde/ 4% (w/v) sucrose, and permeabilized with 0.2% (v/v) Triton X-100 in phosphate-buffered saline followed by incubation with primary antibodies and fluorophore-conjugated secondary antibodies.

### Image analysis and quantification

Images captured by confocal microscopy (LSM 810, Zeiss, Gottingen, Germany) were analyzed blindly using MetaMorph software (Version: 7.10.1.161, Molecular Devices, San Jose, CA, USA). Sholl analysis of dendritic arbors was performed employing the modified method of Nakayama et al.^43^. After images of individual neurons were printed, printouts were placed under a clear sheet featuring concentric circles with diameters increasing in 20 µm increments. The center of the circles was placed at the cell body center and the numbers of dendrites crossing each concentric circle were counted. Quantification of primary and secondary dendrites was also performed by imaging of individual neurons. The density of dendritic spine and synaptic protein clusters were measured from 27 to 32 dendrites of 7 ~ 9 neurons.

### Statistical analysis

GraphPad Prism software (GraphPad Software, Inc., La Jolla, CA, USA) was used to perform all statistical analyses. Two-sample comparisons were conducted with Student’s t-tests, while multiple comparisons were performed with a one-way analysis of variance (ANOVA) followed by Tukey post hoc tests. All results are presented as the means ± s.d. or means ± s.e.m. Differences with a *p* value less than 0.05 were considered to be statistically significant.

## Supplementary Information

Supplementary Information.
