# Publisher Correction: Nanoplasmonic immunosensor for the detection of SCG2, a candidate serum biomarker for the early diagnosis of neurodevelopmental disorder

**DOI:** 10.1038/s41598-021-03378-6

**Published:** 2021-12-16

**Authors:** So-Hee Lim, Yun-Ju Sung, Narae Jo, Na-Yoon Lee, Kyoung-Shim Kim, Da Yong Lee, Nam-Soon Kim, Jeehun Lee, Ju-Young Byun, Yong-Beom Shin, Jae-Ran Lee

**Affiliations:** 1 Rare Disease Research Center, Korea Research Institute of Bioscience and Biotechnology, 125 Gwahak-ro, Yuseong-gu, Daejeon, 34141 Korea; 2 BioNanotechnology Research Center, Korea Research Institute of Bioscience and Biotechnology, 125 Gwahak-ro, Yuseong-gu, Daejeon, 34141 Korea; 3 BioNano Health Guard Research Center (H-GUARD), 125 Gwahak-ro, Yuseong-gu, Daejeon, 34141 Korea; 4 Department of Bio-Molecular Science, KRIBB School of Bioscience, Korea University of Science and Technology (UST), 217 Gajeong-ro, Yuseong-gu, Daejeon, 34113 Korea; 5 Laboratory Animal Resource Center, Korea Research Institute of Bioscience and Biotechnology, 125 Gwahak-ro, Yuseong-gu, Daejeon, 34141 Korea; 6 Department of Pediatrics, Samsung Medical Center, Sungkyunkwan University School of Medicine, 81 Irwon-ro, Gangnam-gu, Seoul, 06351 Korea

Correction to: *Scientific Reports* https://doi.org/10.1038/s41598-021-02262-7, published online 23 November 2021

In the original version of this Article So-Hee Lim, Yun-Ju Sung, Narae Jo and Na-Yoon Lee were omitted as equally contributing authors.

The original Article has been corrected.

