# Supplementary material for: Nanoplasmonic immunosensor for the detection of SCG2, a candidate serum biomarker for the early diagnosis of neurodevelopmental disorder
Source: Sci Rep. 2021 Nov 23;11:22764. doi: 10.1038/s41598-021-02262-7 (PMC8610996; doi:10.1038/s41598-021-02262-7)
Supplement: Supplementary file 1 — Supplementary Information. [file 41598_2021_2262_MOESM1_ESM.pdf]

Supplementary Information

Figure S1. Production of anti-SCG2 antibody. Anti-SCG2 antibody was produced against human SCG2 peptide as an antigen in rabbit. The homemade SCG2 antibody detected SCG2 protein from rat brain to a level comparable to that obtained with a commercial SCG2 antibody (A). In addition, the homemade SCG2 antibody worked well for immunoblotting and immunoprecipitation of recombinant human SCG2 protein expressed in heterologous cells (B). SCG2-AB, commercial anti-SCG2 antibody; SCG2-HM, home-made anti-SCG2 antibody; hSCG2, human SCG2 expression construct. The original images are presented in Fig. S1-1.

Fig S1

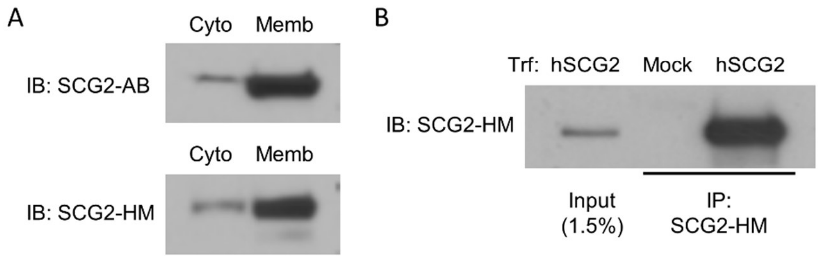

Figure S1-1. Production of anti-SCG2 antibody. These are the original images of Fig. S1.

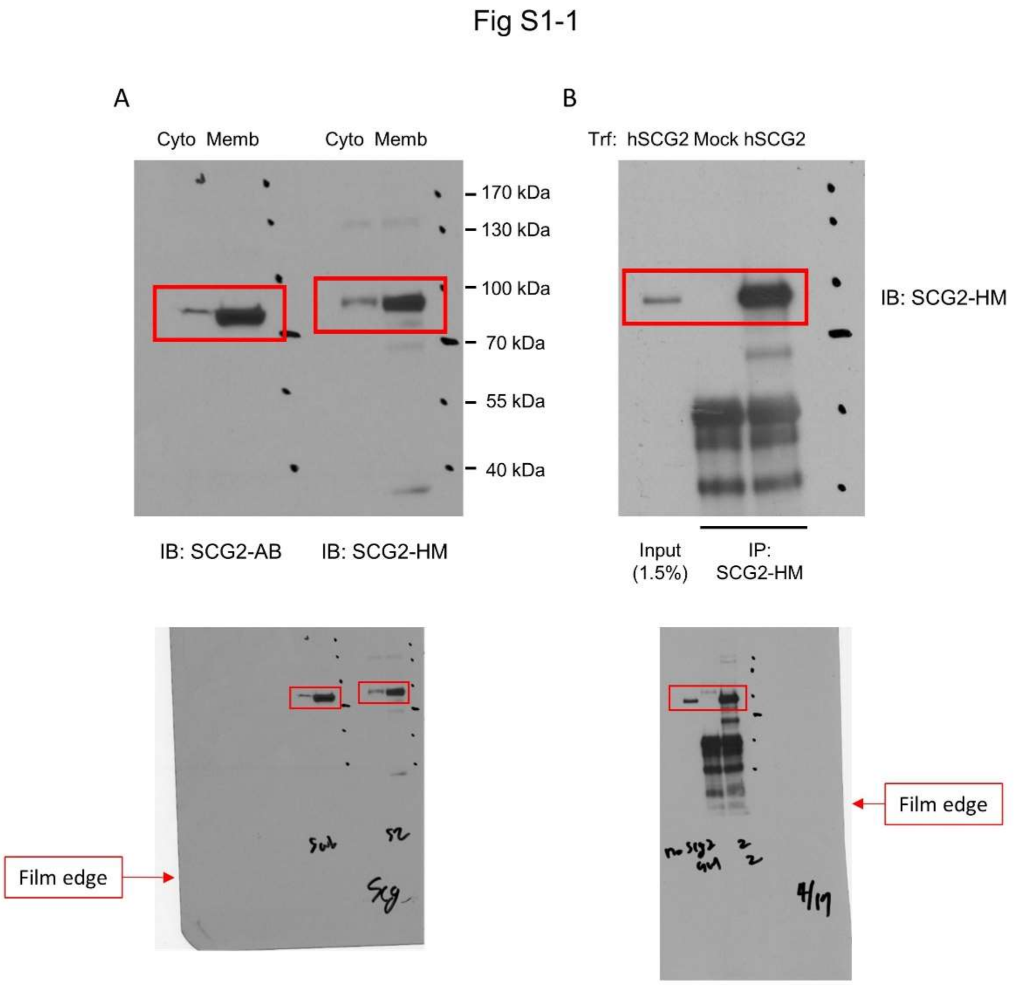

Figure S2. Analytical performance of conventional ELISA for SCG2 detection. (A) Optimization of concentration of SCG detection antibody in conventional ELISA. SCG2 (125 ng/mL) was analyzed for condition optimization. (B) Standard curve was prepared by plotting the absorbance measured with varying concentration of SCG2 (from 0 to 125 ng/mL). The regression equation was y = 0.0031x + 0.093 (R^2 = 0.9911) in the linear range. Each data point is the everage of N=3 individual measurements, and the error bars indicate standard deviation.

Fig S2

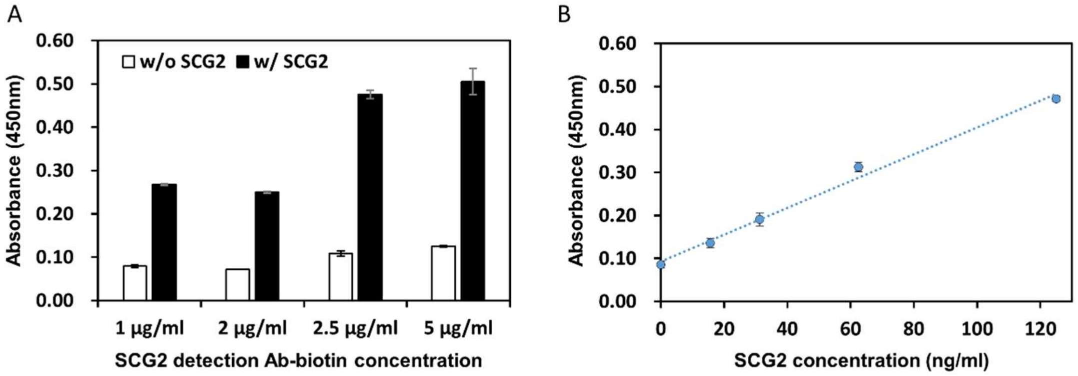

Figure S3. Sensor chip preparation. Schematic represents the self-assembly monolayer formation of 11-MUA on gold nanodot in conjunction with immobilization of antibodies.

Fig S3

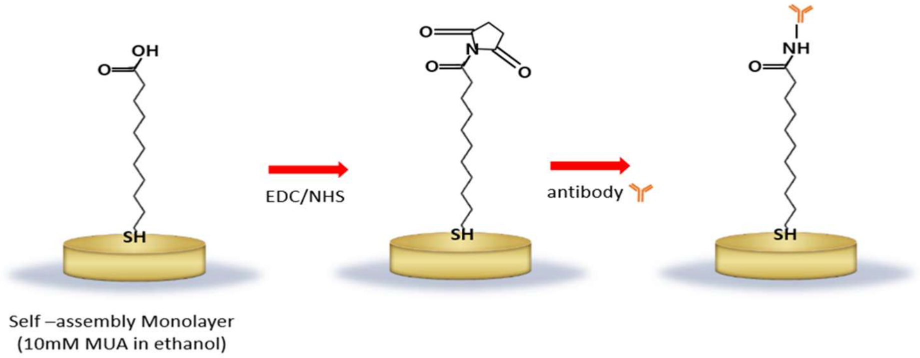

Figure S4. Optimization of enhanced nanoplasmonic immunosensor. (A) Optimization of blocking buffer. The assay was performed in the absence of SCG2. BSA, bovine serum albumin. (B) Optimization of concentration of biotinylated anti-SCG2 antibody (Ab). The assay was performed using 10 ng/mL SCG2. $\Delta\lambda$ was defined as the shift in LSPR wavelength between the before and after the NBT-BCIP reaction. $\Delta\lambda_{control}$: LSPR peak shift in the absence of SCG2, $\Delta\lambda_{sample}$: LSPR peak shift in the presence of SCG2. All experiments were repeated three times, and the error bars represent the standard deviations of the LSPR wavelength shifts.

Fig S4

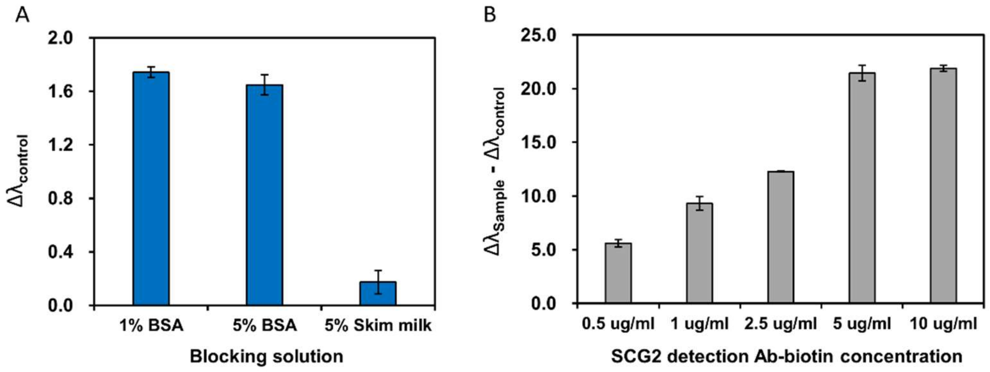

Figure S5. Specificity analysis of enhanced plasmonic immunosensor. The concentrations of SCG2 and other interfering substance were 15 ng/mL in 10% human serum. IL-6, interleukin-6; PSA, prostate-specific antigen; AFP, alpha fetoprotein; NC, negative control without analytes. All experiments were repeated three times, and the error bars represent the standard deviations of the LSPR wavelength shifts.

Fig S5

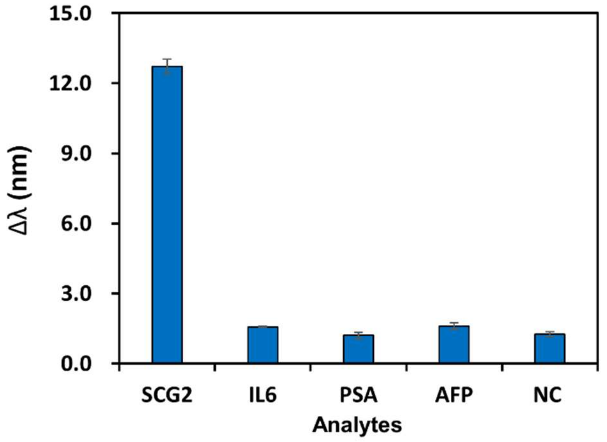

Figure S6. Decreased SCG2 in the brain tissues of *PTPRT*$^{-/-}$ null mice. SCG2 expression was examined in the cortical and hippocampal tissues of *PTPRT*$^{-/-}$ null mice. These are the original images of Fig. 5A. The representative protein bands have been selected from western blotting of seven *PTPRT*$^{+/+}$ wild-type mice and six *PTPRT*$^{-/-}$ null mice for Fig. 5A. We compared the ratio of SCG2 levels relative to β-actin between wild-type and null mice. The numbers of mice were displayed above each band of SCG2 and β-actin.

Fig S6

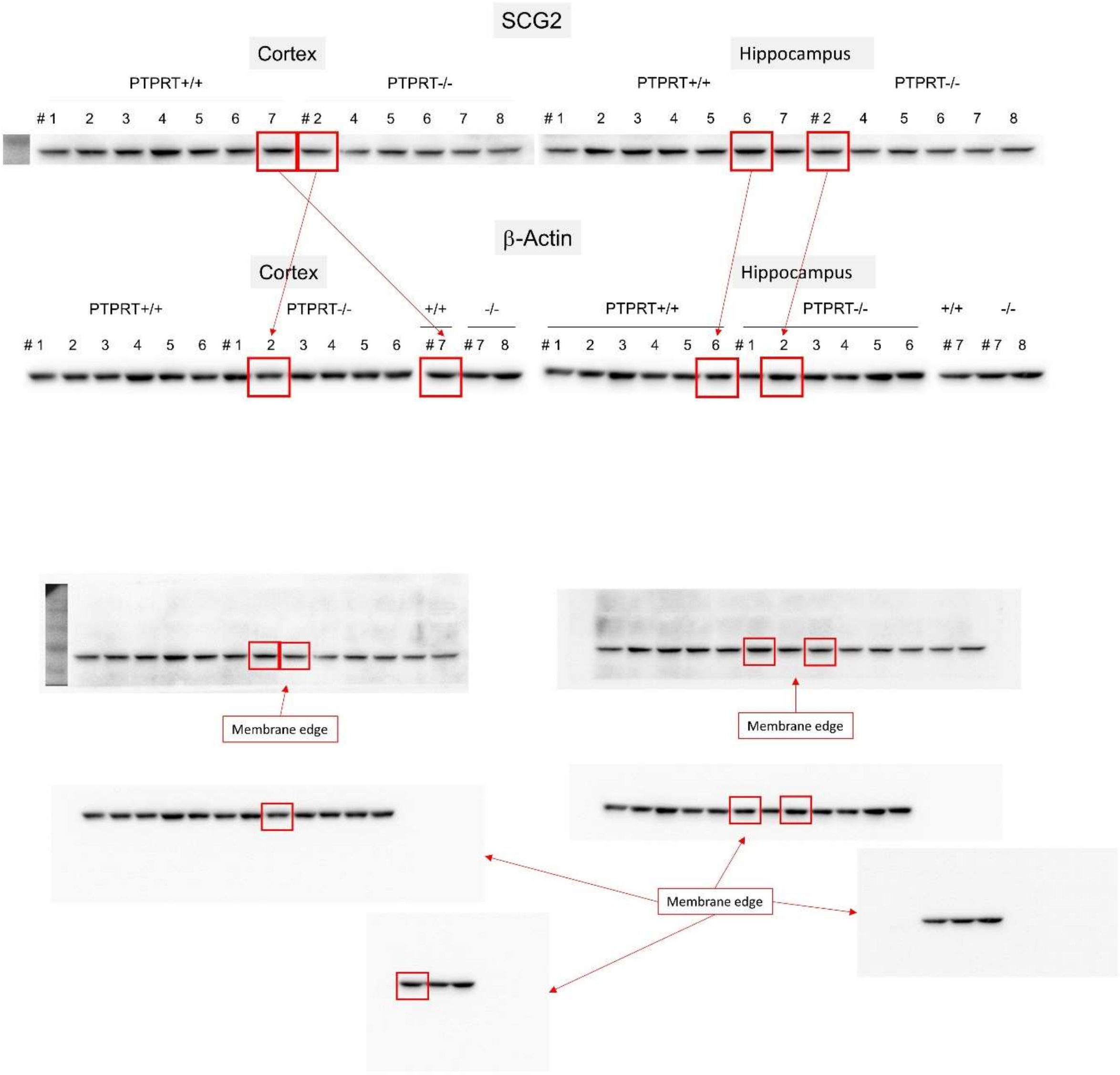

Figure S7. shRNA for knockdown of rat SCG2 and rescue SCG2 construct. pSuper.gfp/neo (OligoEngine, Seattle, WA, USA) was used for construction of shRNA for knockdown of rat SCG2. (A) When rat SCG2 and SCG2-shRNA were co-transfected in HEK cells, SCG-shRNA effectively knocked down SCG2 expression. (B) Res SCG2 expression was shown to be resistant to SCG2-shRNA. The original images are presented in Fig. S7-1.

Fig S7

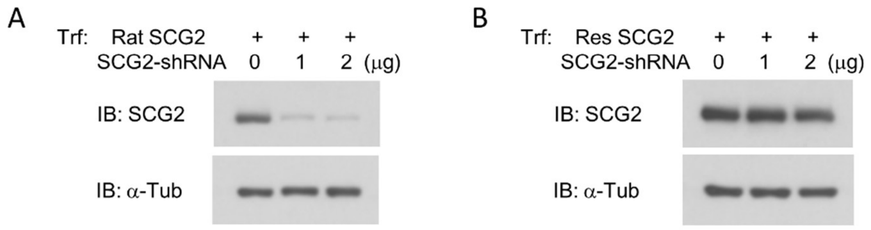

Figure S7-1. shRNA for knockdown of rat SCG2 and rescue SCG2 construct. These are the original images of Fig. S7.

Fig S7-1

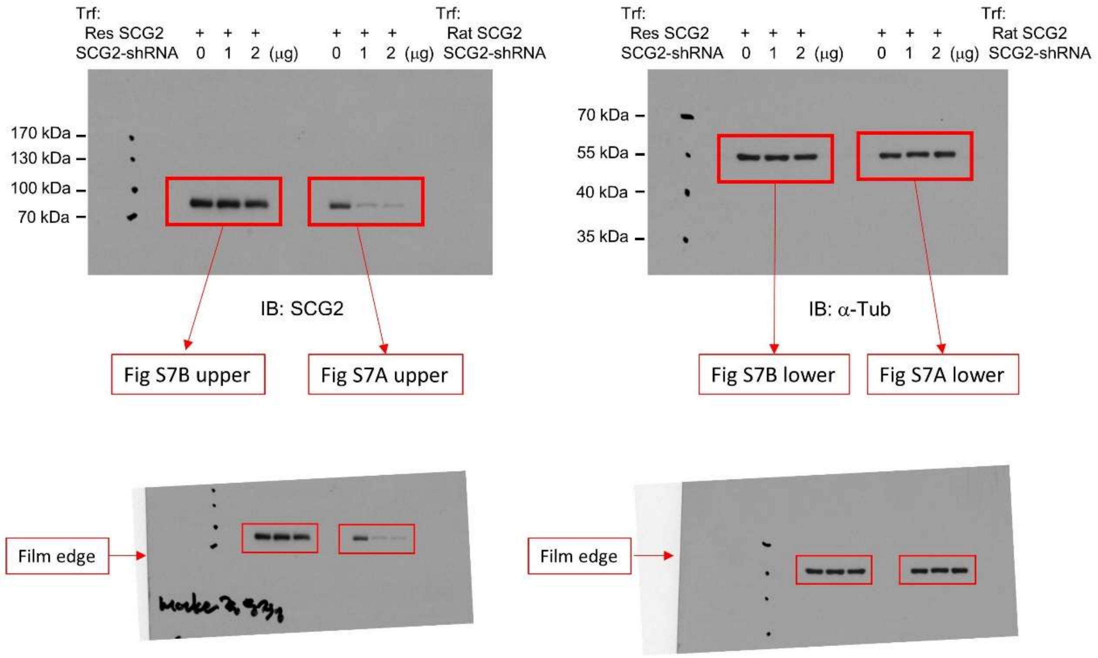

Table S1. Basic information of patients and control groups. For E-NPIS analysis, serum samples of eight patients with developmental delay and five controls were used. On the other hand, serum samples of six patients and two controls were used in the ELISA analysis because SCG2 concentration of some samples was below the LOD of ELISA. DD, developmental delay; CT, control; M, male; F, female.

Table S1

Sample #	Sex	Age	E-NPIS	ELISA
DD1	F	0 Years 3 Months	o	o
DD2	F	1 Years 3 Months	o	o
DD3	M	1 Years 6 Months	o	o
DD4	F	1 Years 8 Months	o	o
DD5	F	1 Years 9 Months	o	o
DD6	F	2 Years 3 Months	o	
DD7	M	5 Years 11 Months	o	o
DD8	F	8 Years 9 Months	o	
CT1	M	2 Years 0 Months	o	o
CT2	F	4 Years 0 Months	o	
CT3	M	10 Years 8 Months	o	o
CT4	F	11 Years 0 Months	o	
CT5	F	14 Years 0 Months	o	

Table S2. The performance of E-NPIS compared to other LSPR based immunosensors.

Materials/ nanostructure	Target	enhancement strategy	Detection limit	Ref.
Silver nanostructures	NS1 antigen	Label-free	500 ng/mL	1
Gold Nano rod	AFP, PSA	Label-free	0.5 ng/mL	2
Gold nanodisk	PSA	Label-free	1.7 ng/mL	3
Gold nano dot	α-amylase	Label-free	500 ng/mL	4
Gold nanoparticle	CRP	Label-free	10 ng/mL	5
Gold nanoparticle	Hepatitis B virus antigen	Gold nanoparticle	0.001 ng/mL	6
Gold nanohole/ Gold nanopillar	PSA	Quantum dot	0.1 ng/mL	7
Gold nano island	cTnI. CK-MB	IR-dye	0.01 ng/mL	8
Gold strip	IL-10	Gold nanocube	3.6 ng/ml	9
Gold nanodot	SCG2	Tyramide amplification	0.016 ng/mL	This study

References

1. Pearlson, P. A. S. & Ashis, K. S. Localized surface plasmon resonance (LSPR) biosensor based on thermally annealed silver nanostructures with on-chip blood-plasma separation for the detection of dengue non-structural protein NS1 antigen. *Biosens. Bioelectron.* **132**, 38-46 (2019).
2. Acimovic, S. S., Ortega, M. A., Sanz, V., Berthelot, J., Garcia-Cordero, J. L., Renger, J., Maerkl, S. J., Kreuzer, M., P. & Quidant, R. LSPR Chip for Parallel, Rapid, and Sensitive Detection of Cancer Markers in Serum. *Nano Lett.* **14**, 2636-2641 (2014).
3. Khan, Y. Li, A., Chang, L., Li, L. & Guo, L. Gold nano disks arrays for localized surface plasmon resonance based detection of PSA cancer marker Sens. *Actuators B Chem.* **255,** 1298-1307 (2018).
4. Guerreiro, J. R. L., Frederiksen, M., Bochenkov, V. E., Freitas, V. D., Sales, M. G. F. & Sutherland, D. S. Multifunctional Biosensor Based on Localized Surface Plasmon Resonance for Monitoring Small Molecule-Protein Interaction. *ACS Nano* **8**, 7958–7967 (2014).
5. Oh, S. Y., Heo, N. S., Bajpai, V. K., Jang, S. C., Ok, G., Cho, Y. & Huh, Y. S. Development of a Cuvette-Based LSPR Sensor Chip Using a Plasmonically Active Transparent Strip. *Front. Bioeng. Biotechnol.* **7,** 299 (2019).
6. Kim, J., Oh, S. Y., Shukla, S., Hong, S. B., Heo, N. S., Bajpai, V. K., Chun, H. S., Jo, C. H., Choi, B. G., Huh, Y. S. & Han, Y. K. Heteroassembled gold nanoparticles with sandwich-immunoassay LSPR chip format for rapid and sensitive detection of hepatitis B virus surface antigen (HBsAg). *Biosens. Bioelectron.* **107**, 118-122 (2018).
7. Song, H. Y., Wong, T. I., Sadovoy, A., Wu, L., Bai, P., Deng, J., Guo, S., Wang, Y., Knoll, W. & Zhou, X. Imprinted gold 2D nanoarray for highly sensitive and convenient PSA detection via plasmon excited quantum dots. *Lab Chip* **15,** 253-263 (2015).

8. Xu, W., Wang, L., Zhang, R., Sun, X., Huang, L., Su, H., Wei, X., Chen, C. C., Lou, J., Dai, H. & Qian, K. Diagnosis and prognosis of myocardial infarction on a plasmonic chip. *Nat. Commun*. **11**, 1654 (2020).

9. Baek, S. H., Song, H. W., Lee, S., Kim, J. E., Kim, Y. H., Wi, J. S., Ok, J. G., Park, J. S., Hong, S., Kwak, M. K., Lee, H. J. & Nam, S. W. Gold Nanoparticle-Enhanced and Roll-to-Roll Nanoimprinted LSPR Platform for Detecting Interleukin-10. *Front. Chem.* **8,** 285 (2020).
